# Utility of superoxide dismutase C (*sodC*) gene-based real-time PCR for detecting *Neisseria meningitidis* carriage in pharyngeal swab samples

**DOI:** 10.1128/spectrum.01248-25

**Published:** 2025-07-24

**Authors:** Tesfaye Gelanew, Getachew Tesfaye Beyne

**Affiliations:** 1 Armauer Hansen Research Institute 70605, Addis Ababa, Ethiopia; End TB Dx Consulting LLC, San Diego, California, USA

**Keywords:** *Neisseria meningitidis*, sodC, porA, ctrA, real-time PCR

## LETTER

We wish to address the recent study by Pham-Tran et al. (1), which evaluated the utility of a superoxide dismutase C (*sodC*) gene-based real-time PCR (rt-PCR) assay for detecting *Neisseria meningitidis* (Nm) carriage in pharyngeal swab samples. Their findings, suggesting a poor sensitivity of *sodC*-based rt-PCR as low as 13%, prompt several methodological concerns that warrant clarification.

Globally, bacterial meningitis is primarily caused by Nm, *Streptococcus pneumoniae*, and *Haemophilus influenzae* (Hi) (2), which together account for most reported cases. While the capsule transport operon A (*ctrA*) gene is often targeted in rt-PCR detection of Nm, its susceptibility to rearrangement and absence in up to 16% of carried meningococcal isolates (3) pose limitations. Conversely, *sodC*—located outside the capsule locus—is less prone to antigenic variation, essential for Nm survival, and conserved across all 12 Nm serogroups while absent in other *Neisseria* species (2). These attributes make *sodC* a promising alternative target for Nm detection.

Pham-Tran et al.’s (1) approach to validating their *sodC* assay raises notable issues. Effective assay validation requires the inclusion of well-characterized positive and negative samples confirmed by multiple methods or gold standards. Their reliance on *porA*-based rt-PCR as the sole gold standard is problematic, as *porA* is neither universally present across all Nm isolates nor exclusively specific to Nm, being detected in some other *Neisseria* species (4). Besides, *porA* is prone to deletion via recombination between repetitive elements within the gene (5), which causes false-negative rt-PCR results. Thus, incorporating an independent assay as a tie-breaker would have bolstered their conclusions.

Furthermore, the possibility of co-colonization with pathogens such as *S. pneumoniae* or Hi highlights the need for *sodC*-specific primers and probes optimized to prevent cross-reactivity or false positives. Pham-Tran et al. acknowledged the lack of specificity in their *sodC* probe but did not sufficiently address this issue. Similarly, *porA* qPCR primers and probes cited in the literature exhibit significant mismatches (3, 4), which Pham-Tran et al. (1) could have redesigned and optimized for improved specificity.

Contrary to their findings, several studies have demonstrated high sensitivity and specificity of *sodC*-based assays. Thomas et al. reported detection of 98.9% of culture-positive, nongroupable Nm samples using *sodC*-based rt-PCR with a specificity of 100% across 244 non-Nm isolates. Although the presence of *sodC* in Hib isolates has been documented, Thomas et al. found no cross-reactivity among 88 Hib isolates or 100 culture-positive Brazilian and CHOA Hib carriage specimens tested with Nm *sodC* assays. Additionally, Sirluck-Schroeder et al. validated the specificity of *sodC*-based rt-PCR, showing no cross-reactivity across diverse sample panels, including *Haemophilus influenzae* DNA-positive samples (6).

Sensitivity studies further support *sodC*-based assays, with U.S. studies reporting a sensitivity of 99.6% (518/520) for detecting meningococcal carriage isolates (7). Similarly, our in-house *sodC* assay has consistently demonstrated 100% sensitivity in culture-confirmed Nm samples (8). It is important to note that *sodC* is specific to Nm and was not detected in any other N*eisseria* species, which makes it one of the best markers for distinguishing Nm (3), particularly in carriage status.

We assert that the conclusions of Pham-Tran et al. (1) are misleading due to methodological shortcomings, including poor primer specificity and inadequate optimization. A comprehensive validation against a broader panel of bacterial species, particularly *S. pneumoniae* and Hib, is essential to ensure robust assay performance. Given the vital role of accurate Nm detection in effective surveillance and public health interventions, we urge caution in interpreting the clinical utility of rt-PCR assays for Nm. Rigorous evaluation of assay validation methods, performance sample panels, and primer/probe designs is crucial, particularly for detecting nasopharyngeal carriage in adults.

## EDITOR'S NOTE

**Ed. Note**: The authors of the original article declined to respond.
